# Myelin Oligodendrocyte Glycoprotein Antibody-Associated Disease and Varicella Zoster Virus Infection - Frequency of an Association 

**DOI:** 10.3389/fimmu.2021.769653

**Published:** 2021-10-19

**Authors:** Franziska Di Pauli, Paul Morschewsky, Klaus Berek, Michael Auer, Angelika Bauer, Thomas Berger, Gabriel Bsteh, Paul Rhomberg, Kathrin Schanda, Anne Zinganell, Florian Deisenhammer, Markus Reindl, Harald Hegen

**Affiliations:** ^1^ Department of Neurology, Medical University of Innsbruck, Innsbruck, Austria; ^2^ Department of Neurology, Medical University of Vienna, Vienna, Austria; ^3^ Department of Neuroradiology, Medical University of Innsbruck, Innsbruck, Austria

**Keywords:** AQP-4-IgG, MOG-IgG, MOG antibody associated diseases, longitudinally extensive transverse myelitis, varicella zoster virus infection, neuromyelitis optica spectrum disorder

## Abstract

To determine whether there is a correlation between myelin oligodendrocyte glycoprotein (MOG) antibody-associated diseases and varicella zoster virus (VZV) infection. We provide a case report and performed a study to determine the frequency of MOG antibodies (MOG-IgG) in neurological VZV infections. Patients admitted to the Medical University of Innsbruck from 2008–2020 with a diagnosis of a neurological manifestation of VZV infection (n=59) were included in this study; patients with neuroborreliosis (n=34) served as control group. MOG-IgG was detected using live cell-based assays. In addition, we performed a literature review focusing on MOG and aquaporin-4 (AQP4) antibodies and their association with VZV infection. Our case presented with VZV-associated longitudinally extensive transverse myelitis and had MOG-IgG at a titer of 1:1280. In the study, we did not detect MOG-IgG in any other patient neither in the VZV group (including 15 with VZV encephalitis/myelitis) nor in the neuroborreliosis group. In the review of the literature, 3 cases with MOG-IgG and additional 9 cases with AQP4 IgG associated disorders in association with a VZV infection were identified. MOG-IgG are rarely detected in patients with VZV infections associated with neurological diseases.

## 1 Introduction

Varicella zoster virus (VZV) is an exclusively human neurotropic herpes virus, presents with chickenpox (varicella) as inaugural infection, and remains latent in the dorsal root ganglions, cranial nerves, and the autonomic nervous system, and upon reactivation, results in rash and pain in one or more dermatomes, known as shingles (herpes zoster). This would occur often decades after the primary infection, particularly in susceptible immunocompromised patients, and older patients due to immunosenescence (1).

Typical VZV neurological complications include postherpetic neuralgia, VZV vasculopathy, cranial nerve neuropathy, and radiculopathy (2). Central nervous system (CNS) demyelinating-inflammatory diseases, such as myelitis and encephalitis, are rare complications in primary VZV infection and VZV reactivation (3, 4). It has been suggested that VZV myelitis is caused by direct viral invasion of the spinal cord and cell lysis (5). However, there is mounting evidence for an immune-mediated mechanism, as there are several case reports of aquaporin-4 antibody (AQP4-IgG) positive neuromyelitis optica spectrum disorder (NMOSD) following VZV infection (6–13). Recently, in addition to AQP4-IgG associated NMOSD, the identification of myelin oligodendrocyte glycoprotein (MOG) antibodies has broadened the spectrum of antibody-associated CNS demyelinating-inflammatory diseases that are distinct from classical multiple sclerosis (14). Similar to the identification of AQP4-IgG following VZV infection, some case reports described MOG-IgG antibody-associated disease (MOGAD) following VZV, influenza A, or herpes simplex virus infection (15–18).

However, until now, there has been no systematic analysis about the association of MOG-IgG and neurological manifestations of VZV infection. Here, we describe a patient with MOG-IgG positive VZV-associated longitudinally extensive transverse myelitis (LETM), perform a study to determine the MOG-IgG frequencies in patients with VZV infection and neurological involvement and present the results of a literature review.

## 2. Materials and Methods

### 2.1 Patients and Samples

The retrospective study included 59 patients who were admitted to the Medical University of Innsbruck between 2008 and 2020 with the diagnosis of a neurological manifestation due to VZV infection and had an available serum sample of at least 500 µl. Diagnosis of VZV infection with neurological involvement (i.e., meningitis, encephalitis, myelitis, encephalomyelitis, cranial nerve or/and segmental zoster paresis) was based on the presence of typical dermatomal rash followed by neurological symptoms and supported by laboratory findings (elevated CSF cell count, positive VZV DNA in the cerebrospinal fluid (CSF) as determined by polymerase chain reaction (PCR), or increased CSF VZV-IgG) (19, 20). CNS involvement was defined as encephalitis or myelitis or a combination of both. In the absence of a typical rash, diagnosis was always based on a positive CSF VZV DNA and CSF pleocytosis.

Patients with neuroborreliosis [previously published (21)] were included as control group (n=34), as this is also a disease entity of infectious origin that might affect the CNS as well as the PNS, and also typically shows elevated CSF cell count and disrupted blood-CSF-barrier as indicated by elevated Qalb. In addition, there is no known association of neuroborreliosis with AQP4 or MOG antibodies. Briefly, these patients were admitted to Medical University of Innsbruck between 2009 and 2016 and received diagnosis of neuroborreliosis according to EFNS criteria (22). Diagnosis was based on typical neurological symptoms, appropriate routine CSF findings (pleocytosis, blood-CSF barrier impairment, and/or intrathecal synthesis of immunoglobulins), and intrathecal synthesis of borrelia-specific IgG antibodies [antibody specificity index (ASI) >1.5] (22).

Results of routine diagnostic procedures, clinical, magnetic resonance imaging (MRI), and CSF data were collected. Routine CSF work-up comprised red blood cell (RBC) and white blood cell (WBC) count, CSF total protein concentration, CSF/serum albumin quotient, and CSF and serum IgG, IgM, and IgA concentrations. Intrathecal synthesis of IgG, IgM and IgA were calculated by the Auer and Hegen formula (23) and expressed as percentage intrathecal fraction. IgG index was calculated as [CSF IgG/serum IgG]/[CSF albumin/serum albumin]. CSF was collected by lumbar puncture and blood by simultaneously peripheral venous puncture. Serum was isolated from blood by centrifugation after the blood samples were allowed to clot for ≥30 min. All samples were centrifuged at 2000 g for 10 min at room temperature.

### 2.2 MOG-IgG Assay

The presence of MOG-IgG was determined by live cell-based immunofluorescence assay with HEK293 cells transfected with full-length human MOG (alpha-1 isoform), as previously described (24). Screening for serum antibodies was performed at 1:20 and 1:40 dilutions by two independent investigators blinded for the clinical diagnosis. An isolated IgM reactivity was excluded by the use of heavy chain-specific secondary antibodies against IgG (Dianova, Hamburg, Germany) (24).

### 2.3 Ethics

The study was approved by the Ethics Committee of the Medical University of Innsbruck (approval number AM3041A). Written informed consent was obtained from all patients. Authorization has been obtained for disclosure (consent to disclose) from the index case patient.

### 2.4 Statistics

Statistical analysis was performed using SPSS 26.0 (SPSS Inc, Chicago, IL, USA). Non-parametric data were displayed as median and interquartile range. Categorical variables were reported as frequency and percentage. For group comparisons, Mann-Whitney-U and χ^2^ tests were applied, as appropriate. Two-sided P-values <0.05 were considered statistically significant.

### 2.5 Literature Review

We conducted a literature search in MEDLINE and Google Scholar. Search terms were: VZV AND MOG or AQP4 or NMOSD or LETM; herpes zoster AND MOG or AQP4 or NMOSD or LETM. Abstracts that primarily did not deal with VZV infection and MOGAD or NMOSD or LETM were excluded. In addition, articles identified in reference lists of the individual papers were selected if considered appropriate.

## 3 Results

### 3.1 Index Case

**Figure 1:**
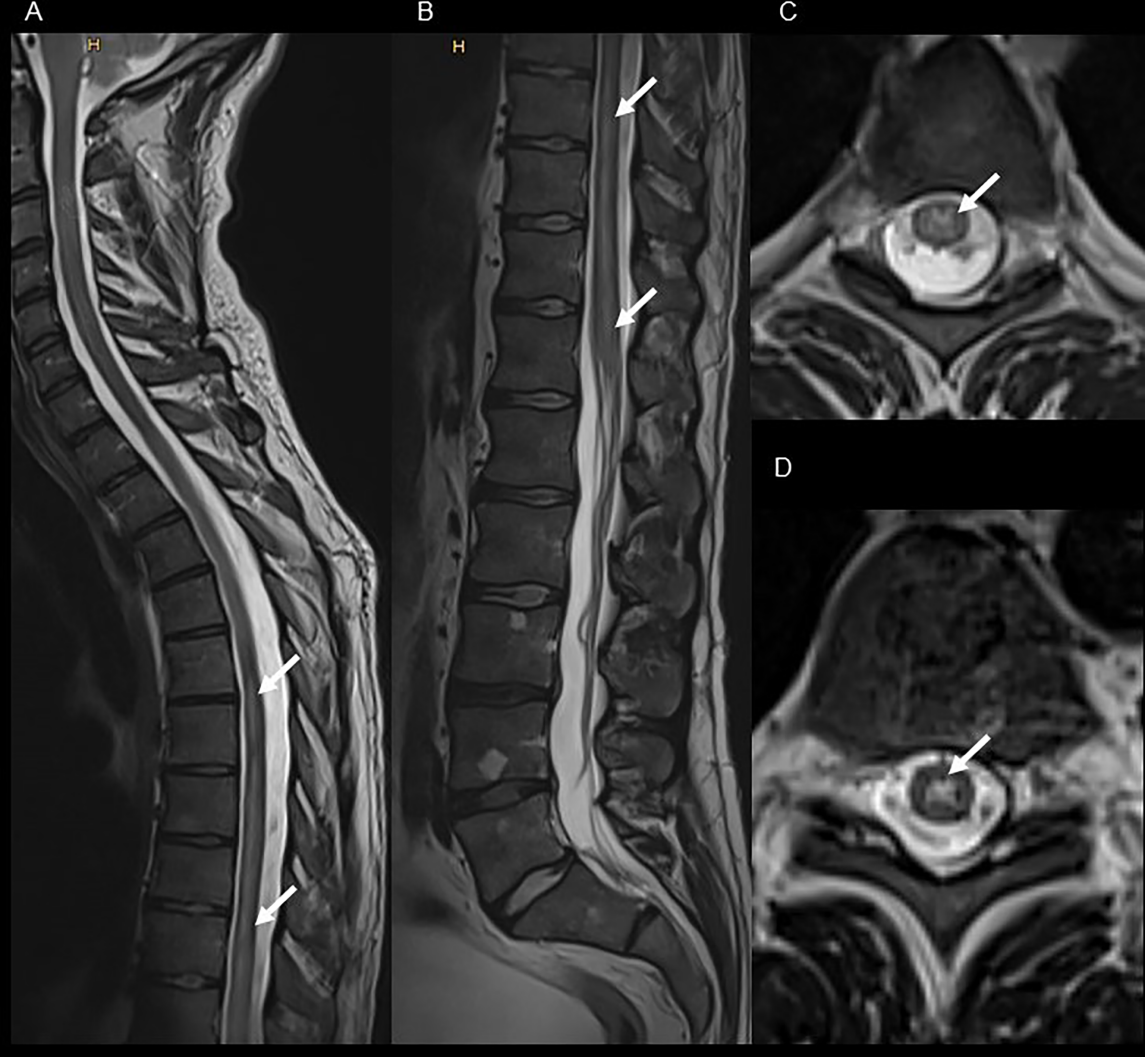
Spinal MRI of the index patient presenting with MOG-IgG associated LETM following VZV infection. T2 weighted cervical, thoracic [panel **(A)**, sagittal view] and lumbar [panel **(B)**, sagittal view] spinal cord MRI shows extensive hyperintense lesion from the level T1 to the conus medullaris [panel **(C)**, axial view of T4; and panel **(D)**, axial view of T9] with only a very faint contrast enhancement (not shown).

A 30-year-old, previously healthy man presented in 2019 with sensomotor paralytic syndrome (sensory level below T6), subsequent gait ataxia and neurogenic bladder disturbance requiring catheterization at our emergency department. Six days before the first neurological symptoms, the patient had developed herpes zoster infection (dermatome T6 right side) treated by his general practitioner with oral acyclovir (5 days, 3 x 1000 mg per day orally). There was no history of constitutional symptoms or a recent vaccination. A MRI of the spinal cord showed a T2 hyperintense lesion extending from T1 to conus medullaris confined to gray matter (**Figure 1**) with only a very faint contrast enhancement, whereas brain MRI was normal. CSF analysis revealed lymphocytic pleocytosis with a WBC count of 101 cells/μl, and oligoclonal bands were negative. Despite the VZV DNA PCR results being negative, the CSF VZV antibody-specific index (ASI) was highly elevated (9.4). However, in addition, MOG-IgG in serum were positive at high titer (1:1280), while AQP4-IgG were absent. Further diagnostic work-up to determine immune deficiency or a malignancy was negative (including a whole body 18F-fluorodeoxyglucose positron emission tomography-computed tomography, human immunodeficiency virus screening, serum immunoglobulin levels, flow cytometry of peripheral blood). MOG-IgG associated LETM following VZV infection was diagnosed, and the patient was treated with a combination of high-dose methylprednisolone (10 days: 1,000 mg for 3 days, 500mg for 4 days, 250 mg for 3 days) followed by oral tappering and intravenous acyclovir (3 x 750 mg for 10 days, 3 x 500 mg for 8 days followed by oral acyclovir 3 x 1000 mg for 5 days). Thereafter, no further disease-modifying or immunosuppressive therapy was started. After three months, the MOG-IgG titer had decreased to 1:320, and MOG-IgG was undetectable after another five months. Except for mild neurogenic bladder dysfunction, there was complete clinical and imaging remission without further relapses after an 18-month follow-up. EDSS improved from an initial score of 3.5 to 1.0 at 18-month follow-up.

### 3.2 Retrospective Study

#### 3.2.1 Demographic, Clinical, and Main Cerebrospinal Fluid Characteristics

**Figure 2:**
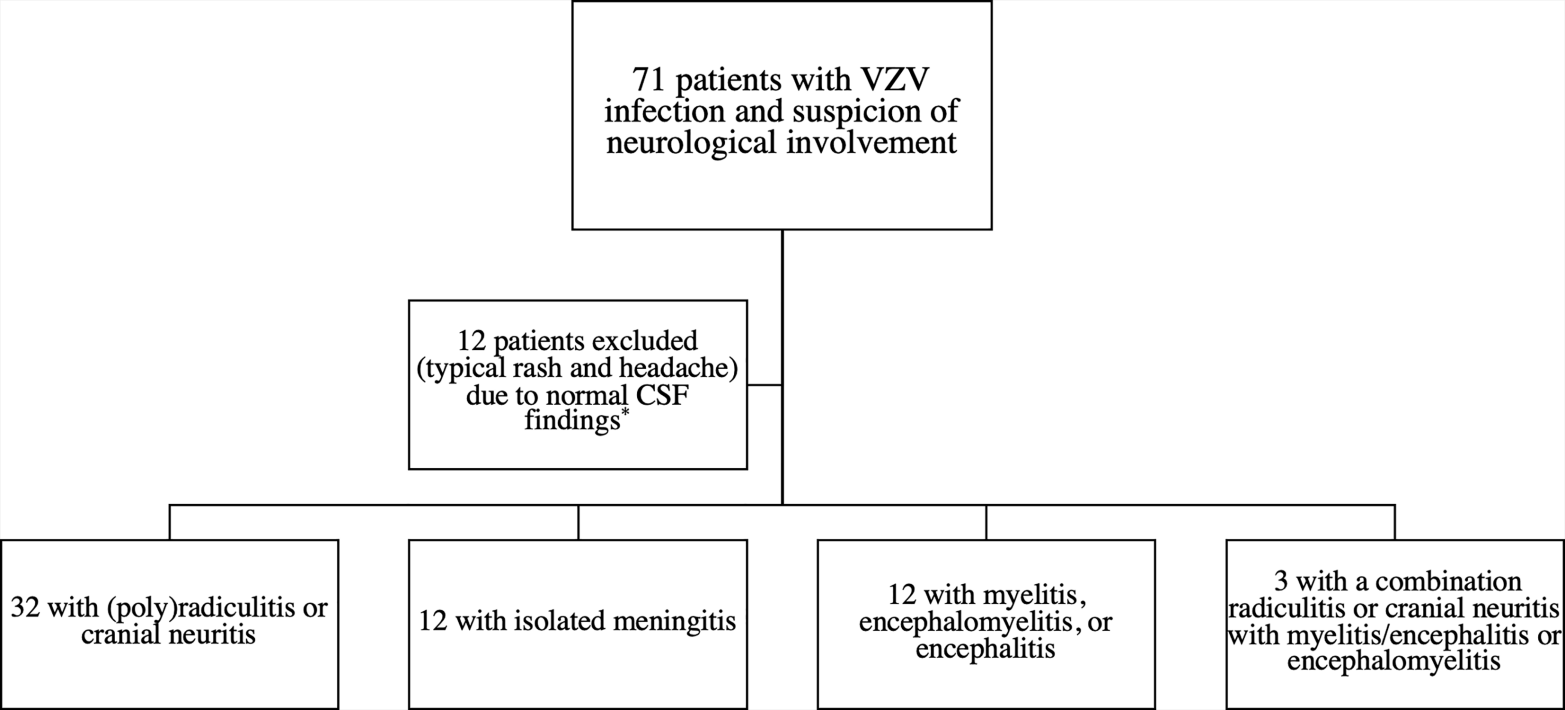
Inclusion flowchart VZV infection with neurological involvement. * Negative CSF VZV specific antibody index, VZV DNA PCR and normal CSF cell count. CSF, cerebrospinal fluid; VZV, varicella zoster virus.

A total of 59 patients with neurological involvement due to VZV infection and 34 patients with neuroborreliosis were included into this study (**Figure 2**). Demographic and main clinical characteristics of patients with VZV infection and CNS involvement are shown in **Table 1**, those of patients with neuroborreliosis elsewhere (21). Fifteen patients with a VZV infection (25.4%) presented with either myelitis (n=6; one patient with a LETM), encephalomyelitis (n=1), or encephalitis (n=8). Parenchymal CNS involvement occurred in three (8.8%) of the patients with neuroborreliosis. Patients with neuroborreliosis (median age 46 years, interquartile range [IQR] 10.4–65.6) were younger than patients with VZV infection (median age 63 years, IQR 45.5–76, p<0.001), while males and females were equally distributed in both disease groups (p=0.610). The average interval between typical VZV-associated rashes and neurological symptoms was 7 days (range 2-12). In **Table 2**, the main CSF findings of VZV infection with CNS involvement and neuroborreliosis are shown. In both diseases, the WBC count was increased, although the WBC count was significantly higher in patients with neuroborreliosis. Intrathecal IgG and IgM fraction (%) was significantly elevated in patients with neuroborreliosis compared to patients with VZV infection.

**Table 1:** Demographic and clinical data of patients with varicella zoster virus infection with central nervous system involvement.

Sex (female), n (%)	28 (47.5%)
Age (years), median (IQR)	63 (45.5–76)
**Clinical presentation, n (%)**	
(Poly)radiculitis or cranial neuritis	32 (54.2%)
Isolated meningitis	12 (20.3%)
Myelitis, encephalomyelitis, or encephalitis	12 (20.3%)
Combination radiculitis or cranial neuritis with myelitis/encephalitis or encephalomyelitis	3 (5.1%)
Days between rash and neurological symptoms, median (IQR)	7 (2–12)
Typical VZV associated rash, n (%)	53 (89.8%)
**Diagnosis based on, n (%)**	
Positive VZV DNA PCR	44 (74.6%)
Typical clinical features and elevated CSF cell count	3 (5.1%)
Increased CSF VZV IgG	12 (20.3%)

VZV, varicella zoster virus; IQR, interquartile range; n, number.

**Table 2:** Cerebrospinal fluid characteristics of patients with varicella zoster virus infection and neuroborreliosis.

	VZV infection	Neuroborreliosis	p-value^a^
RBC count (cells/μL), median (IQR)	2 (0–21)	4 (0–7)	0.719
WBC count (cells/μL), median (IQR)	99 (18–274)	154 (105–216)	0.037
CSF/serum glucose ratio, median (IQR)	0.55 (0.48–0.67)	0.53 (0.44–0.65)	0.227
CSF total protein (mg/dL), median (IQR)	67 (46–102)	102 (46–179)	0.132
Qalb, median (IQR)	9.44 (6.79–16.22)	14.79 (6.8–28.83)	0.183
IgG index, median (IQR)	0.58 (0.51–0.66)	0.79 (0.62–1.01)	<0.001^*^
Intrathecal IgG synthesis (%), median (IQR)^1^	0 (0–0)	2.02 (0–25.56)	<0.001^*^
Intrathecal IgA synthesis (%), median (IQR) ^1^	0 (0–0)	0 (0–6.39)	0.059
Intrathecal IgM synthesis (%), median (IQR) ^2^	0 (0–0)	42.54 (0–66.4)	<0.001^*^
VZV antibody index, median (IQR)^3^	2.1 (0.6–3.1)	na	na
Borrelia antibody index, median (IQR)	na	23.6 (7.5–43.8)	na

Data from: ^1^ 90 cases, ^2^ 85 cases, ^3^ 31 cases (18 cases>1.5). CSF WBC and RBC were counted within the Fuchs-Rosenthal chamber (volume of 3.2 μL). Counts are reported as “cells/μL” (correction for a standard volume of 1 μL was achieved by dividing by 3.2). CSF, cerebrospinal fluid; IQR, interquartile range; n, number; na, not applicable; PCR, polymerase chain reaction; RBC, red blood cell; WBC, white blood cell; Qalb, CSF/serum albumin quotient; VZV, varicella zoster virus.

a calculated by Mann-Whitney U test, uncorrected p-values are shown, after Bonferroni correction still significant at a level of 0.05.

#### 3.2.2 MOG-IgG in VZV Infection With CNS Involvement

All patients – those with VZV infection with CNS involvement and those with neuroborreliosis – were negative for serum MOG-IgG. One patient with VZV infection and radiculitis had a borderline MOG-IgG positive titer of 1:160. This result was not confirmed by using heavy chain–specific secondary antibodies and was therefore regarded as negative.

### 3.3 Literature Review

We identified 2 case reports (15, 25) and 1/10 patients in a case series (20) with MOG antibody-associated myelitis in association with a VZV infection; in addition, there are 9 reports of AQP4 antibody-associated CNS disorders in patients with VZV infections (6–13, 20) (**Table 3**).

**Table 3:** Varicella zoster virus infection-associated cases with MOG/AQP4 antibody associated central nervous system disorders.

Case	Clinical phenotype	Neurological symptoms	Presence of rash	Time of onset after Rash in days	Level rash	VZV DNA PCR	Presence/absence of VZV-IgG/IgM elevation	Imaging findings	Antibody status	Follow-up	Reference#
**Cases with MOG IgG**											
Male, 69 a	LETM	Paraparesis	+	10	Left L2-3 dermatome	+	nk	Lesion from the bottom of the medulla oblongata to the upper (T2) thoracic region	MOG-IgG +, cell-based-assay		(15)
Female, 34 a	Myelitis	Paraparesis, loss of pain and temperature sensation below her groin, absent vibration sense in both lower limbs	primary VZV infection	21	na	nd	nd	normal	MOG-IgG +	Clinical remission	(25)
										MOG IgG remained positive	
Female, 42 a	LETM	nk	+	1	C dermatome	nk	Increased VZV IgM	Myelitis: C2-4,T 1, Medulla oblongata	MOG-IgG +	Clinical remission (EDSS 6 to 1), relapse, NMOSD criteria fulfilled	(20)
Male, 30 a	LETM	Sensomotor paralytic syndrome (sensory level below T6), subsequent gait ataxia, neurogenic bladder disturbance	+	6	Right T6 dermatome	–	CSF VZV ASI increased (9.4)	Lesion: T1 to the conus medullaris with only a very faint leptomeningeal contrast enhancement	MOG-IgG + (1:1280), cell-based assay	Clinical remission	Present case
										MOG IgG turned negative	
**Cases with AQP4 IgG**											
Female, 63 a	LETM	Paresis (3–4/5) and mild hypoesthesia of the left leg, sensory impairment for temperature and pain of the right leg and the trunk below level T10, urine incontinence	+	14	Along the lumbar spine	–	CSF VZV ASI normal	Lesion from C7 to Th9 with marked oedema and moderate gadolinium enhancement	AQP4-IgG +, tissue-based indirect immunofluorescence assays	Partial clinical remission (after plasmapheresis)	(7)
										AQP-4 IgG turned negative	
Female, 51 a	LETM,	Decreased power (3/5), hyperreflexia along with sensory loss in the right upper and lower extremity, hyperesthesia in the entire left lower extremity	+	49	Right C5 dermatome	–	nk	Enhancing intramedullary lesion C2 -4, centrally into the right of the midline with signal changes at the T1 level without enhancement or expansive appearance	AQP4-IgG first attack nd, relapse + (>1:160)	Two relapses, diagnosis NMOSD, persistent AQP-4 IgG, clinical remission	(8)
Female, 59 a	LETM	nk	+	15	C dermatome	nk	Increased VZV IgM	Myelitis: C1-6	AQP4-IgG +	Clinical remission (EDSS 2 to 1), no relapse, NMOSD criteria fulfilled	(20)
Female, 29 a	LETM	Acute quadriplegia	+	7	Left T4–6 dermatomes	–	Increased VZV IgM	nk	AQP4-IgG first attack nd, relapse + (1:80), tissue-based indirect immunofluorescence assays	Partial clinical remission, relapse - LETM, NMOSD criteria fulfilled	(9)
Female, 77 a	LETM	Paraparesis, sensory level by L4, urine retention	+	2	Left L4–S1 dermatomes	+	nk	Lesion extending from C2–C3 to T12 with no gadolinium enhancement	AQP4-IgG first attack nd, relapse + indirect immunofluorescence serum assay (1:10)	Severe sequelae, relapse, NMOSD criteria fulfilled	(10)
Female, 48 a	LETM	Right arm abduction paresis, brisk reflexes in the lower limbs, diminished reflexes in the upper limbs, extensor plantar response bilaterally	+	14	Right C6 dermatome	–	nk	Cervical LETM	AQP4-IgG positive, cell-based assay	Fully recovered, except for mild sensory symptoms, NMOSD criteria fulfilled	(11)
Female, 53 a	LETM	Hyperhidrosis of left side of her face, neck, arm and upper chest, muscle weakness of her left leg, sensory impairment for light touch and temperature in her chest and legs	+	7	T5-6 dermatome	nk	CSF VZV IgG index increased (7.9)	Lesion extending from T1-7	AQP4-IgG +	Relapse	(12)
Female, 55 a	LETM	Dysesthesia of the right side of the face, neck, bilateral upper extremities, and T4-T10 levels, urine incontinence	+	14	Left C3-T4 dermatomes	–	CSF VZV ASI increased (4.53)	Lesion extending from the lower part of the medulla oblongata to C5, with marked edema and moderate gadolinium enhancement and abnormal gadolinium enhancement of the left spinal posterior root	AQP4-IgG +, cell-based assay	Mild response to treatment, relapse, NMOSD criteria fulfilled	(6)
Female, 17 a	Area postrema syndrome and LETM	Right eye mydriasis, piloerection, poikilothermia, mild hypoesthesia, and pain in the right arm and trunk in the T2-T3 dermatomes/intractable vomiting	+	21	Right T2 dermatome	–	IgM VZV ASI increased (7.0)	Lesions involved the area postrema, right ventrothalamic area, periaqueductal gray, optic tracts, and cervical and thoracic regions, longitudinally extended from C1-5 and from C6-T6 and axially involving two-thirds of the spinal cord	AQP4-IgG +	NMOSD criteria fulfilled, resolution	(13)

A, age; AQP4, aquaporin-4; ASI, antibody specific index; C, cervica; CSF, cerebrospinal fluid; LETM, longitudinally extensive transverse myelitis; MOG, myelin oligodendrocyte glycoprotein; L, lumbar; MRI, magnetic resonance imaging; not applicable, na; nd, not done; NMOSD, neuromyelitis optica spectrum disease; nk, not known; T, thoracic; VZV, varicella zoster virus; +, positive; -, negative.

## 4 Discussion

Here, we present a case with MOG-IgG associated LETM triggered by VZV infection. In a subsequent retrospective study of 59 patients with VZV infection and neurological involvement; however, we did not found MOG-IgG in any patient (including 15 with VZV encephalitis/myelitis).

MOG-IgG are more often present in children than in adults and are associated with a variable clinical spectrum. Typical clinical presentation of MOGAD, particularly in children, is an acute disseminated encephalomyelitis (approximately 50%), whereas in adults myelitis (up to 30%) or optic neuritis (up to 50%) are more common (14, 26–28). Similar to our case, MOG-IgG associated myelitis is characterized in the MRI by longitudinally extensive T2 hyperintense lesions affecting mainly the grey matter and lack of contrast enhancement (29). MOG-IgG case reports showing an association between MOGAD and VZV infection are rare. Two case reports described the occurrence of LETM after herpes zoster and chicken pox, respectively (15, 25) (**Table 3**). In only one patient, the VZV DNA PCR result was available and reported as positive. In our case, the VZV DNA PCR result was negative, which may have been due to the preceding acyclovir therapy (6 days). However, elevated WBC count and a highly increased VZV ASI confirmed the diagnosis. In a group of 10 immunocompetent patients with VZV infection–related myelitis, MOG-IgG was present in one patient (20). This patient relapsed and fulfilled the seronegative NMOSD criteria during follow-up. In contrast, our patient showed nearly complete recovery without further relapses, and MOG-IgG was undetectable after eight months. Approximately 35% of patients with MOGAD have a relapsing disease (14). Although persistent MOG-IgG positivity is only a moderate marker for relapsing disease with a positive predictive value of approximately 60%, a conversion to undetectable antibody reliably predicted a monophasic disease course in approximately 90% of cases (14). Due to limitations such as a lack of prospective clinical trials in MOGAD and established standard test criteria conversion of detectable to undetectable MOG-IgG is currently not a reliable marker for treatment decisions. However, as a) only approximately one third of the patients with MOG-IgG relapse, b) MOG-IgG was negative during follow-up, and c) due to the known viral trigger, we decided not to start a disease modifying or immunosuppressive treatment. In addition to MOG-IgG, several case reports have described an association of AQP4-IgG with VZV infection (**Table 3**), we identified 9 further reports of AQP4-IgG associated CNS inflammatory demyelinating disorder related to a VZV infection. In synopsis with the MOG-IgG associated cases, eleven patients out of twelve presented with LETM (the one remaining case was MRI-negative myelitis), LETM seems to be a typical clinical presentation of these rare associations. Of particular importance, for eight patients, there was information available that indicated that the NMOSD (1/8 MOG-IgG positive, 7/8 AQP4-IgG positive) criteria were fulfilled, and that relapses occurred in at least six patients. Therefore, in the rare clinical presentation of LETM triggered by a VZV infection, screening for MOG-IgG or AQP4-IgG has therapeutic implications. In contrast to cases in whom MOG-IgG are detected, treatment with a disease-modifying therapy should be considered if AQP4-IgG is detected.

The pathophysiological basis of the association between VZV infection and MOG or AQP4 antibody-associated associated LETM is unclear. In the majority of the reported cases, CSF VZV DNA PCR results were negative (**Table 3**), and a direct viral invasion of the spinal cord seems unlikely, although pathological data are missing. These data are consistent with the retrospective case series of immunocompetent individuals with VZV myelitis published by Wang et al. (20). Four out of 11 patients fulfilled the NMOSD criteria, and despite immunosuppressive treatment in the two relapsing patients, no VZV reactivity was observed. Given the rising number of cases with the presence of MOG-IgG or AQP4-IgG and the typically delayed onset of neurological symptoms (**Table 3**) after the rash, immune-mediated genesis seems likely. The mechanisms suspected to be involved in triggering autoimmunity after infection are molecular mimicry, bystander activation, epitope spreading, and the release of cryptic antigens (30). A possible hypothesis for MOG and AQP4 antibody-associated autoimmunity triggered by a VZV infection is that the VZV infection causes a breakdown of the blood-brain barrier, as indicated by the common finding of an elevated CSF/serum albumin ratio in herpes zoster (31). Subsequently, CNS antigen is released into the periphery, which induces an immune reaction against self-antigens by autoreactive B and T cells.

A limitation of our study is the retrospective design and small number (n=15) of patients with myelitis, encephalomyelitis, or encephalitis. However, data specifically excluded a nonspecific bystander reaction, as patients with VZV infection without parenchymal involvement were negative for MOG-IgG (n=44). As CSF analysis of MOG-IgG improve the sensitivity by 7%, another limitation is that MOG-IgG was only tested in patient sera (32).

Overall, we showed that the presence of MOG-IgG is a rare finding in patients with a VZV infection complicated by CNS demyelinating-inflammatory diseases. Nevertheless, due to therapeutic implications, antibody screening is a useful tool, particularly in patients with a higher pre-test probability, e.g. with LETM. Further prospective larger studies, including children, are required to analyze the frequency of neurological antibody-associated diseases triggered by VZV infection.

## Data Availability Statement

The raw data supporting the conclusions of this article will be made available by the authors, without undue reservation.

## Ethics Statement

The studies involving human participants were reviewed and approved by Medical University Innsbruck. Written informed consent to participate in this study was provided by the participants’ legal guardian/next of kin. Written informed consent was obtained from the individual(s) for the publication of any potentially identifiable images or data included in this article.

## Author Contributions

FDP conceptualized the study, collected data, case analysis, statistical analysis, drafted the manuscript, and revised the manuscript for intellectual content. PM collected data and revised the manuscript for intellectual content. KB collected data and revised the manuscript for intellectual content. MA collected data and revised the manuscript for intellectual content. AB collected data and revised the manuscript for intellectual content. TB case analysis and revised the manuscript for intellectual content. GB collected data and revised the manuscript for intellectual content. PR MRI analysis and revised the manuscript for intellectual content. KS collected data and revised the manuscript for intellectual content. AZ collected data and revised the manuscript for intellectual content. FDe case analysis, conceptualized the study, and revised the manuscript for intellectual content. MR conceptualized the study, and revised the manuscript for intellectual content. HH conceptualized the study, collected data, case analysis, statistical analysis, drafted the manuscript, and revised the manuscript for intellectual content. All authors contributed to the article and approved the submitted version.

## Funding

This study was funded by a research grant from the Austrian Science Fund (FWF projects P32699, MR).

## Conflict of Interest

FDP has participated in meetings sponsored by, received honoraria (lectures, advisory boards, consultations) or travel funding from Almirall, Bayer, Biogen, Celgene, Janssen, Merck, Novartis, Sanofi-Genzyme, Roche and Teva. Her institution has received research grants from Roche. KB has participated in meetings sponsored by and received travel funding from Roche. MA received speaker honoraria and/or travel grants from Biogen, Merck, Novartis and Sanofi. AB has participated in meetings sponsored by Merck and Biogen. TB has participated in meetings sponsored by and received honoraria (lectures, advisory boards, consultations) from pharmaceutical companies marketing treatments for multiple sclerosis: Almirall, Biogen, Biologix, Bionorica, Celgene/BMS, GSK, MedDay, Merck, Novartis, Roche, Sandoz, Sanofi/Genzyme, TG Pharmaceuticals, TEVA-ratiopharm and UCB. His institution has received financial support in the last 12 months by unrestricted research grants (Biogen, Bayer, Celgene/BMS, Merck, Novartis, Roche, Sanofi/Genzyme, and TEVA ratiopharm) and for participation in clinical trials in multiple sclerosis sponsored by Alexion, Bayer, Biogen, Merck, Novartis, Roche, Sanofi/Genzyme, and TEVA. GB has participated in meetings sponsored by, received speaker honoraria or travel funding from Biogen, Celgene, Lilly, Merck, Novartis, Roche, Sanofi-Genzyme and Teva, and received honoraria for consulting Biogen, Celgene, Roche and Teva. AZ has participated in meetings sponsored by, received speaking honoraria or travel funding from Biogen, Merck, Sanofi-Genzyme and Teva. FDe has participated in meetings sponsored by or received honoraria for acting as an advisor/speaker for Almirall, Alexion, Biogen, Celgene, Genzyme-Sanofi, Merck, Novartis Pharma, Roche, and TEVA ratiopharm. His institution has received research grants from Biogen and Genzyme Sanofi. He is section editor of the MSARD Journal (Multiple Sclerosis and Related Disorders). MR was supported by a research support from Euroimmun and Roche. His institution receives payments for antibody assays (MOG, AQP4, and other autoantibodies) and for MOG and AQP4 antibody validation experiments organized by Euroimmun (Lübeck, Germany). HH has participated in meetings sponsored by, received speaker honoraria or travel funding from Bayer, Biogen, Merck, Novartis, Sanofi-Genzyme, Siemens, Teva, and received honoraria for acting as consultant for Biogen and Teva.

The remaining authors declare that the research was conducted in the absence of any commercial or financial relationships that could be construed as a potential conflict of interest.

## Publisher’s Note

All claims expressed in this article are solely those of the authors and do not necessarily represent those of their affiliated organizations, or those of the publisher, the editors and the reviewers. Any product that may be evaluated in this article, or claim that may be made by its manufacturer, is not guaranteed or endorsed by the publisher.

## References

[1] Arvin A. Aging, Immunity, and the Varicella-Zoster Virus. N Engl J Med (2005) 352:2266–7. doi: 10.1056/NEJMp058091 15930416

[2] Kennedy PGE Gershon AA. Clinical Features of Varicella-Zoster Virus Infection. Viruses (2018) 10:609. doi: 10.3390/v10110609 PMC6266119 30400213

[3] Nagel MA Gilden D. Neurological Complications of Varicella Zoster Virus Reactivation. Curr Opin Neurol (2014) 27:356–60. doi: 10.1097/WCO.0000000000000092 PMC4189810 24792344

[4] Bozzola E Tozzi AE Bozzola M Krzysztofiak A Valentini D Grandin A. Neurological Complications of Varicella in Childhood: Case Series and a Systematic Review of the Literature. Vaccine (2012) 30:5785–90. doi: 10.1016/j.vaccine.2012.05.057 22683522

[5] Hogan EL Krigman MR. Herpes Zoster Myelitis. Evidence Viral Invasion Spinal Cord Arch Neurol (1973) 29:309–13. doi: 10.1001/archneur.1973.00490290049004 4355263

[6] Eguchi H Takeshige H Nakajima S Kanou M Nakajima A Fuse A. Herpes Zoster Radiculomyelitis With Aquaporin-4 Antibodies: A Case Report and Literature Review. Front Neurol (2020) 11:585303. doi: 10.3389/fneur.2020.585303 33329330 PMC7719747

[7] Heerlein K Jarius S Jacobi C Rohde S Storch-Hagenlocher B Wildemann B. Aquaporin-4 Antibody Positive Longitudinally Extensive Transverse Myelitis Following Varicella Zoster Infection. J Neurol Sci (2009) 276:184–6. doi: 10.1016/j.jns.2008.08.015 18805556

[8] Jayarangaiah A Sehgal R Epperla N. Sjögren’s Syndrome and Neuromyelitis Optica Spectrum Disorders (NMOSD)–A Case Report and Review of Literature. BMC Neurol (2014) 14:200. doi: 10.1186/s12883-014-0200-5 25291981 PMC4193162

[9] Park JS Hwang SJ Shin JH Kim DS. A Recurrent Longitudinally Extensive Transverse Myelitis With Aquaporin-4(AQP4) Antibody After Herpes Zoster. J Neurol Sci (2013) 334:69–71. doi: 10.1016/j.jns.2013.07.2510 23953947

[10] Machado C Amorim J Rocha J Pereira J Lourenço E Pinho J. Neuromyelitis Optica Spectrum Disorder and Varicella-Zoster Infection. J Neurol Sci (2015) 358:520–1. doi: 10.1016/j.jns.2015.09.374 26440423

[11] Mathew T Thomas K Shivde S Venkatesh S Rockey SM. Post Herpes Zoster Infection Neuromyelitis Optica Spectrum Disorder. Mult Scler Relat Disord (2017) 18:93–4. doi: 10.1016/j.msard.2017.09.022 29141830

[12] Suda M Tsutsumiuchi M Uesaka Y Hayashi N. A Case of Anti Aquapolin-4 Antibody Positive Myelitis With Hyperhidrosis, Following Herpes Zoster. Rinsho Shinkeigaku (2017) 57:26–8. doi: 10.5692/clinicalneurol.cn-000820 28025408

[13] Turco EC Curti E Maffini V Pisani F Granella F. Neuromyelitis Optica Spectrum Disorder Attack Triggered by Herpes Zoster Infection. Mult Scler Int (2020) 2020:6151258. doi: 10.1155/2020/6151258 32373365 PMC7196994

[14] Hegen H Reindl M. Recent Developments in MOG-IgG Associated Neurological Disorders. Ther Adv Neurol Disord (2020) 13:1756286420945135. doi: 10.1177/1756286420945135 33029200 PMC7521831

[15] Shiga Y Kamimura T Shimoe Y Takahashi T Kaneko K Kuriyama M. Anti-Myelin Oligodendrocyte Glycoprotein (MOG) Antibody-Positive Varicella-Zoster Virus Myelitis Presenting as Longitudinally Extensive Transverse Myelitis: A Case Report. Rinsho Shinkeigaku (2017) 57:579–83. doi: 10.5692/clinicalneurol.cn-001066 28954973

[16] Amano H Miyamoto N Shimura H Sato DK Fujihara K Ueno S. Influenza-Associated MOG Antibody-Positive Longitudinally Extensive Transverse Myelitis: A Case Report. BMC Neurol (2014) 14:224. doi: 10.1186/s12883-014-0224-x 25434485 PMC4256916

[17] Vieira JP Sequeira J Brito MJ. Postinfectious Anti-Myelin Oligodendrocyte Glycoprotein Antibody Positive Optic Neuritis and Myelitis. J Child Neurol (2017) 32:996–9. doi: 10.1177/0883073817724927 28820014

[18] Nakamura M Iwasaki Y Takahashi T Kaneko K Nakashima I Kunieda T. A Case of MOG Antibody-Positive Bilateral Optic Neuritis and Meningoganglionitis Following a Genital Herpes Simplex Virus Infection. Mult Scler Relat Disord (2017) 17:148–50. doi: 10.1016/j.msard.2017.07.023 29055448

[19] Hung CH Chang KH Kuo HC Huang CC Liao MF Tsai YT. Features of Varicella Zoster Virus Myelitis and Dependence on Immune Status. J Neurol Sci (2012) 318:19–24. doi: 10.1016/j.jns.2012.04.017 22564884

[20] Wang X Zhang X Yu Z Zhang Q Huang D Yu S. Long-Term Outcomes of Varicella Zoster Virus Infection-Related Myelitis in 10 Immunocompetent Patients. J Neuroimmunol (2018) 321:36–40. doi: 10.1016/j.jneuroim.2018.05.005 29957386

[21] Hegen H Milosavljevic D Schnabl C Manowiecka A Walde J Deisenhammer F. Cerebrospinal Fluid Free Light Chains as Diagnostic Biomarker in Neuroborreliosis. Clin Chem Lab Med (2018) 56:1383–91. doi: 10.1515/cclm-2018-0028 29648995

[22] Mygland A Ljøstad U Fingerle V Rupprecht T Schmutzhard E Steiner I. EFNS Guidelines on the Diagnosis and Management of European Lyme Neuroborreliosis. Eur J Neurol (2010) 17:8–16.e1–4. doi: 10.1111/j.1468-1331.2009.02862.x 19930447

[23] Auer M Hegen H Zeileis A Deisenhammer F. Quantitation of Intrathecal Immunoglobulin Synthesis - a New Empirical Formula. Eur J Neurol (2016) 23:713–21. doi: 10.1111/ene.12924 26806360

[24] Reindl M Schanda K Woodhall M Tea F Ramanathan S Sagen J. International Multicenter Examination of MOG Antibody Assays. Neurology(R) Neuroimmunol Neuroinflamm (2020) 7:e674. doi: 10.1212/NXI.0000000000000674 PMC7051197 32024795

[25] Viswanathan LG. Post-Varicella Anti-Myelin Oligodendrocyte Glycoprotein Antibody-Associated Magnetic Resonance Imaging-Negative Myelitis. Clin Exp Neuroimmunol (2021) 12:122–3. doi: 10.1111/cen3.12618

[26] Di Pauli F Berger T. Myelin Oligodendrocyte Glycoprotein Antibody-Associated Disorders: Toward a New Spectrum of Inflammatory Demyelinating CNS Disorders? Front Immunol (2018) 9:2753. doi: 10.3389/fimmu.2018.02753 30555462 PMC6281762

[27] Jarius S Paul F Aktas O Asgari N Dale RC de Seze J. MOG Encephalomyelitis: International Recommendations on Diagnosis and Antibody Testing. J Neuroinflamm (2018) 15:134. doi: 10.1186/s12974-018-1144-2 PMC5932838 29724224

[28] Wynford-Thomas R Jacob A Tomassini V. Neurological Update: MOG Antibody Disease. J Neurol (2019) 266:1280–6. doi: 10.1007/s00415-018-9122-2 PMC6469662 30569382

[29] Dubey D Pittock SJ Krecke KN Morris PP Sechi E Zalewski NL. Clinical, Radiologic, and Prognostic Features of Myelitis Associated With Myelin Oligodendrocyte Glycoprotein Autoantibody. JAMA Neurol (2019) 76:301–9. doi: 10.1001/jamaneurol.2018.4053 PMC6440233 30575890

[30] Ercolini AM Miller SD. The Role of Infections in Autoimmune Disease. Clin Exp Immunol (2009) 155:1–15. doi: 10.1111/j.1365-2249.2008.03834.x 19076824 PMC2665673

[31] Haanpää M Dastidar P Weinberg A Levin M Miettinen A Lapinlampi A. CSF and MRI Findings in Patients With Acute Herpes Zoster. Neurology (1998) 51:1405–11. doi: 10.1212/WNL.51.5.1405 9818869

[32] Mariotto S Gajofatto A Batzu L Delogu R Sechi G Leoni S. Relevance of Antibodies to Myelin Oligodendrocyte Glycoprotein in CSF of Seronegative Cases. Neurology (2019) 93:e1867–72. doi: 10.1212/WNL.0000000000008479 31645473

